# A Fact for Our Horticultural Readers

**Published:** 1854-12

**Authors:** 


A Fact for our Horticultural Readers.—It has been discovered that for the generality of flowers, and more especially, for geraniums, and the most delicate specimens of the lily tribe, *common glue*, diluted with a sufficient portion of water, forms a richer manure than guano or any other yet discovered. Plants placed in sand on the worst soils, display more beauty and vigor when watered with this composition, than those grown in the richest mould and only sprinkled with water.

